# Hashimoto’s Encephalopathy: A Case Report and Literature Review of an Encephalopathy With Many Names

**DOI:** 10.7759/cureus.9601

**Published:** 2020-08-07

**Authors:** Joseph M DeBiase, Deepti Avasthi

**Affiliations:** 1 Internal Medicine, St. Vincent Mercy Medical Center, Toledo, USA

**Keywords:** hashimoto’s, hashimoto’s encephalopathy, hashimoto’s thyroiditis, autoimmune encephalopathy, encephalopathy

## Abstract

We present the case of a male patient, initially treated for myxedema coma secondary to Hashimoto’s thyroiditis, who was discharged on levothyroxine and a low-dose steroid taper but was re-admitted for the treatment of status epilepticus. During the second admission, the patient developed encephalopathy and cognitive dysfunction. Thyroid peroxidase (TPO) antibodies (Abs) were elevated and the patient was treated with high-dose steroids with clinical improvement. The patient was determined to have Hashimoto’s encephalopathy (HE) due to the clinical picture as well as the response to high-dose glucocorticoid therapy. Cerebrospinal fluid (CSF) analysis demonstrated elevated protein, immunoglobulin G (IgG) index, and IgG synthesis rate; however, albumin index was elevated, indicating a disrupted blood-brain barrier. We suggest that HE be considered in the differential diagnosis for patients presenting with seizures, coma, stroke-like symptoms, behavior changes, and unexplained encephalopathy. After ruling out more common pathologies, HE should be considered by testing for anti-TPO Abs.

## Introduction and background

A 65-year-old Caucasian male initially presented to the emergency department with a chief complaint of altered mental status (AMS), confusion, and memory loss for five days. His family reported an episode of unresponsiveness earlier that day. The patient was noted to be bradycardic and hypotensive with the electrocardiogram (EKG) showing episodes of junctional rhythm. He had another episode of unresponsiveness with subsequent hypoxemia and was therefore intubated with pressor support with admission to the intensive care unit. Complete blood count, basic metabolic profile, head CT, chest X-ray (CXR), echocardiogram, urine drug screen (UDS), random cortisol, and infectious workup were negative. Thyroid-stimulating hormone (TSH) was elevated at 92.76 mIU/L; other results were as follows: T4 free: <0.10 ng/dL, thyroglobulin antibodies (Abs): >3,000 IU/mL, and thyroid peroxidase (TPO) Abs: >1,000 IU/mL (Table 1).

**Table 1:** Summary of admission studies: first admission (total seven days) HD: hospital day; TSH: thyroid-stimulating hormone; Abs: antibodies; MRI: magnetic resonance imaging; EEG: electroencephalogram

Admission no. 1				
	HD 1	HD 3	HD 4	HD 7
TSH	92 mlU/L		59 mIU/L	55 mlU/L
T4 free	<0.10 ng/dL		0.59 ng/dL	0.49 ng/dL
Thyroid peroxidase Abs	1,000 IU/mL			
Thyroglobulin Abs	>3,000 IU/mL			
Imaging/procedures		MRI brain: punctate focus of acute to subacute infarct left pons	EEG: diffuse theta slowing indicating moderate encephalopathy	

**Figure 1:**
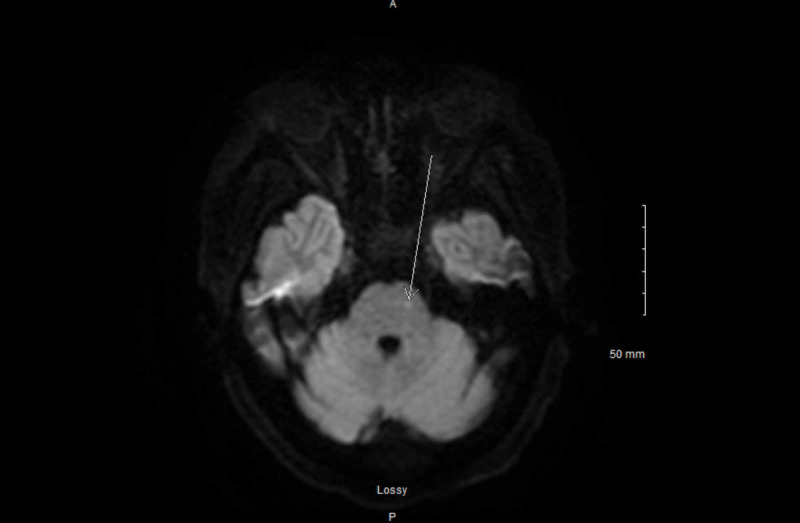
Brain MRI: diffusion-weighted imaging The image shows left pons punctate focus MRI: magnetic resonance imaging

Thyroid ultrasound was not able to visualize thyroid tissue. The patient was loaded with 200 micrograms (mcg) of intravenous (IV) levothyroxine and continued on levothyroxine 100 mcg daily. Liothyronine 10 mcg every eight hours, hydrocortisone 100 milligrams (mg) every eight hours, and levetiracetam 500 mg twice daily were also initiated due to possible seizure-like episodes despite long-term electroencephalography (EEG) showing no epileptic form discharges. Brain MRI showed punctate focus of acute/subacute infarct in the left pons (Figure 1).

On hospital day (HD) four, the patient was extubated and he was recovering well. Ultimately, the patient was discharged on levetiracetam 500 mg twice daily, levothyroxine 200 mcg daily, and prednisone taper from 40 mg to 10 mg over 15 days. Upon discharge, TSH was 55.88 mlU/L, T3 free was 1.69 pg/mL, and T4 free was 0.49 ng/dL.

The patient was again admitted to the hospital two days after discharge for the management of status epilepticus. It was reported that the patient had experienced two episodes of approximately two-minute tonic-clonic seizures with urinary incontinence and post-ictal phase. Labs on arrival were unremarkable except for a TSH of 69.96 mlU/L and T4 free of 1.04 ng/dL (Table 2).

**Table 2:** Summary of laboratory studies/images: second admission* (total 10 days) *Two days following discharge from admission no. 1 HD: hospital day; TSH: thyroid-stimulating hormone; IgG: immunoglobulin G; CSF: cerebrospinal fluid; EEG: electroencephalogram

Admission no. 2					
	HD 1	HD 3	HD 5	HD 7	HD 9
TSH	69 mlU/L	54 mlU/L			5.83 mlU/L
T4 free	1.04 ng/dL	0.84 ng/dL			1.35 ng/dL
IgG, serum			666 mg/dL		
IgG, CSF			34.9 mg/dL		
IgG index, CSF			5.26		
IgG synthesis rate, CSF			157.4 mg/24h		
Albumin index			99.7		
Imaging/procedures		EEG: infrequent sharp transient left temporal/central regions		EEG: frequent bifrontal spike and polyspike wave discharges	EEG: resolution of bifrontal spike wave discharges

EEG on the first day indicated mild diffuse encephalopathy without definite epileptic discharges. The patient was loaded with 1,500 mg levetiracetam and continued on an increased dosage of 1,000 mg twice daily. He was also started on levothyroxine 200 mcg IV, liothyronine 10 mcg IV daily, and the previous dose of prednisone 30 mg daily on HD two. The patient remained seizure-free during hospitalization; however, on HD five, his family voiced concerns about behavior changes. The family noted a “disconnect between his brain and mouth with what he was saying”. On the following day, the patient displayed intermittent confusion, intermittent agitation, repetition of words, inappropriate laughter, staring spells, and displays of odd behaviors such as multiple “epiphanies”. MRI of the brain showed mild chronic small ischemic changes. A lumbar puncture (LP) was performed on HD five. Cerebrospinal fluid (CSF) analysis revealed elevated proteins at 57.7 mg/dL, normal glucose levels, and one white blood cell (WBC). CSF was negative for bacterial or viral etiology, treponema pallidum Ab, West Nile Ab, Lyme Ab, venereal disease research laboratory (VDRL) test, and malignancy. Immunoglobulin G (IgG) CSF synthesis rate was 157.4 mg/24 hours (n=3.3), IgG CSF index: 5.26 (n=<0.70), albumin CSF index: 99.7 (n=<9.0), and negative oligoclonal bands. Serum TPO Abs were 829.0 IU/mL, but the CSF TPO Abs could not be obtained due to the limited volume of CSF. On HD seven, EEG demonstrated frequent bifrontal spikes and polyspike wave discharges, which conferred an increased risk for seizure recurrence. In consultation with neurology, the patient was started on methylprednisolone 250 mg IV (approximately 4 mg/kg prednisone equivalent) every eight hours for the treatment of Hashimoto’s encephalopathy (HE). Neurology tapered levetiracetam off and initiated/increased divalproex to 1,000 mg twice daily over the next couple of HDs as levetiracetam possibly could be contributory to mood changes. Over the course of the next four days, the patient’s mood and agitation improved. Repeat EEG on HD nine (five days after high-dose steroids) showed resolution of previous frequent bifrontal discharges. Ultimately, the patient was discharged on divalproex 1,000 mg twice daily and prednisone 60 mg daily (0.75 mg/kg) with plans to follow up with endocrinology as an outpatient for further glucocorticoid taper. After discharge, the patient has continued to demonstrate clinical improvement.

## Review

The term HE remains controversial. This entity was first described by Lord Brain and colleagues in 1966 pertaining to the case of a 63-year-old male with seizures, disorientation, stroke-like symptoms, and cognitive impairment [1]. Today, there is much debate over its pathogenesis and proper terminology. Castillo et al. favor the term “steroid-responsive encephalopathy associated with autoimmune thyroiditis” (SREAT), and many others believe steroid responsiveness should be considered as a diagnostic criterion for the condition [2]. Caselli et al. described five cases of progressive cognitive decline with psychosis and proposed the term “nonvasculitic autoimmune inflammatory meningoencephalitis” (NAIM) [3]. The estimated prevalence of HE according to one prospective study is 2.1/100,000 people [4].

Terminology remains under scrutiny due to the fact that a clear pathogenesis has not been established by current research. Initially, many theories of direct toxic effect by Abs and releasing hormones were postulated. It was suggested that the direct toxic effect of thyrotropin-releasing hormone (TRH) was the culprit, and this hypothesis stems from the fact that a patient with known HE had worsening myoclonus and tremors with the infusion of TRH [5]. Others postulated direct toxic effect by antithyroid Abs, but there remains little evidence that the Abs have any direct cytotoxic effect in the brain. Ferracci et al. demonstrated antithyroglobulin, antithyroid peroxidase autoantibodies, and circulating immune complexes (CIC) in the CSF of six patients diagnosed with HE. These Abs and CIC were not found in 21 control subjects with different neurological conditions. Interestingly, the elevated Ab indexes but normal albumin index was suggestive of intrathecal synthesis. However, there was a lack of correlation between the clinical evolution of HE and Abs/CIC CSF levels [6]. Some evidence for direct cytotoxic effect was demonstrated by an HE patient’s sera and anti-TPO monoclonal Abs binding to cerebellar astrocytes on monkey brain sections [7].

Another theory of pathogenesis proposed by Forchetti et al. was the disruption of cerebral microvasculature secondary to autoantibody or immune complex deposition evidenced by diffuse and homogenous hypoperfusion on single-photon emission computed tomography (SPECT) [8]. However, current studies have suggested vasculitic pathogenesis [9-10]. Very few post-mortem studies have been done, but, in contrast with the nonvasculitic autoimmune inflammatory meningoencephalitis categorization, brain autopsy of a 77-year-old female with HE provided evidence of lymphocytic infiltrates within leptomeningeal veins and venules, indicating a possible component of vasculitic pathogenesis [11]. CT angiography has also demonstrated vasculitis in HE patients [12].

HE may very well be an under-diagnosed condition due to the various clinical presentations reported to be associated with the condition. Chaudhuri et al. have reported a study involving 18 HE patients. Symptoms of central fatigue (100%), migraine headaches (90%), seizures (67%), stupor/coma (67%), focal neurological deficit (67%), psychosis/delusions/hallucinations (50%), and depression/elevated mood state (10-20%) were reported. Other symptoms included cognitive impairment, alternating hemiparesis, and cerebellar ataxia [13]. Other studies cite behavioral changes in 90-100%, cognitive dysfunction in >80%, and seizure disorder in 60-70% of HE patients. The relapsing and remitting course was suggested in 50% of patients and an insidious progressive course in 40%. The other 10% was not defined [14].

As many conditions may present with encephalopathy and the various symptoms listed above, the diagnosis of HE can be extremely challenging. First and foremost, more common disorders such as sepsis, hepatic encephalopathy, renal failure, structural abnormalities, and vascular/neoplastic disease must be ruled out [14]. Increased anti-thyroid Abs are the most prevalent lab abnormality, and anti-TPO can confirm the diagnosis [14-15]. A Mayo Clinic case series demonstrated that eight out of eight patients were positive for anti-TPO Abs [16]. Hashimoto’s thyroiditis and thyroid hormones cannot be trusted as diagnostic criteria. On a systemic review of the literature relating to 130 patients, euthyroid was diagnosed in 32%, hyperthyroidism/hypothyroidism in 19% each, and subclinical hypothyroidism in 12% of cases [17]. In the same systemic review, high-protein CSF concentration was diagnosed in 71% of patients, and 15 of 20 patients tested for anti-TPO Abs in CSF were positive [16]. Also, 46 out of 101 (45%) patients who underwent MRI had abnormalities, with T2 hyper-signal being the most common abnormality [17]. Newer methods of diagnosis are still being explored. Yoneda et al. have proposed testing for autoantibodies against the amino (NH2)-terminal of α-enolase (NAE) as a useful serological marker [18]. There is no agreed-upon treatment yet, but most treatments include high-dose steroids (prednisone 1-2 mg/kg) with subsequent taper according to symptoms. Corticosteroid duration varies in many cases and can range from four months to 10 years [14]. If glucocorticoids fail, potential second-line therapies include intravenous immunoglobulin (IVIG) [19-20], immunomodulators such as azathioprine or cyclophosphamide, and plasma exchange. Plasma exchange has shown varying treatment results and may not always improve clinical symptoms despite the reduction of TPO Abs [21].

## Conclusions

Our patient demonstrated cognitive impairment, encephalopathy, mood disturbances, and status epilepticus in the setting of Hashimoto’s thyroiditis with elevated anti-TPO Abs. The patient showed marked improvement with high-dose glucocorticoid therapy, leading to the diagnosis of HE. He was initially treated for a myxedema coma with levothyroxine and IV corticosteroids but was ultimately discharged on prednisone taper starting at 0.5 mg/kg. This quick transition to a lower dose corticosteroid treatment may have led to HE presenting with status epilepticus as seen on the second admission. We believe that HE is possibly an under-diagnosed etiology for numerous clinical cases. We recommend that HE should be considered as a differential diagnosis in seizures, stroke-like symptoms, behavioral changes, unexplained delusions or psychosis, and unexplained encephalopathy. After ruling out more common pathologies such as sepsis, bacterial/viral meningitis, hepatic encephalopathy, kidney failure, brain structural abnormality, and neoplasm, HE should be considered in the differential. Our patient demonstrated Hashimoto’s thyroiditis in addition to HE. However, despite thyroiditis, anti-thyroid Abs should be obtained along with CSF anti-thyroid Abs if possible, for a diagnosis of HE. In cases of strong clinical suspicion, high-dose steroids can be initiated, followed by an evaluation of clinical improvement to confirm the diagnosis. Our patient demonstrated elevated CSF protein, IgG CSF synthesis rate, and IgG CSF index as reported in other studies. Despite the elevated IgG CSF synthesis and IgG CSF index, our patient’s albumin index was elevated, indicating a non-intact blood-brain barrier. This could possibly indicate vasculitic pathogenesis. We hope further research will provide more insight into the pathogenesis of HE.
